# Aphids Influence Soil Fungal Communities in Conventional Agricultural Systems

**DOI:** 10.3389/fpls.2019.00895

**Published:** 2019-07-12

**Authors:** Thomas D. J. Wilkinson, Jean-Pascal Miranda, Julia Ferrari, Sue E. Hartley, Angela Hodge

**Affiliations:** ^1^Department of Biology, University of York, York, United Kingdom; ^2^York Environmental Sustainability Institute, University of York, York, United Kingdom

**Keywords:** arbuscular mycorrhizal fungi, community diversity, amplicon sequencing, *Hordeum vulgare*, *Sitobion avenae*, rhizosphere, multitrophic interactions

## Abstract

Arbuscular mycorrhizal fungi (AMF) form symbioses with the roots of most plant species, including cereals. AMF can increase the uptake of nutrients including nitrogen (N) and phosphorus (P), and of silicon (Si) as well as increase host resistance to various stresses. Plants can simultaneously interact with above-ground insect herbivores such as aphids, which can alter the proportion of plant roots colonized by AMF. However, it is unknown if aphids impact the structure of AMF communities colonizing plants or the extent of the extraradical mycelium produced in the soil, both of which can influence the defensive and nutritional benefit a plant derives from the symbiosis. This study investigated the effect of aphids on the plant-AMF interaction in a conventionally managed agricultural system. As plants also interact with other soil fungi, the non-AMF fungal community was also investigated. We hypothesized that aphids would depress plant growth, and reduce intraradical AMF colonization, soil fungal hyphal density and the diversity of AM and non-AM fungal communities. To test the effects of aphids, field plots of barley enclosed with insect proof cages were inoculated with *Sitobion avenae* or remained uninoculated. AMF specific and total fungal amplicon sequencing assessed root fungal communities 46 days after aphid addition. Aphids did not impact above-ground plant biomass, but did increase the grain N:P ratio. Whilst aphid presence had no impact on AMF intraradical colonization, soil fungal hyphal length density, or AMF community characteristics, there was a trend for the aphid treatment to increase vesicle numbers and the relative abundance of the AMF family Gigasporaceae. Contrary to expectations, the aphid treatment also increased the evenness of the total fungal community. This suggests that aphids can influence soil communities in conventional arable systems, a result that could have implications for multitrophic feedback loops between crop pests and soil organisms across the above-below-ground interface.

## Introduction

Arbuscular mycorrhizal fungi (AMF) form obligate symbioses with the roots of c. two-thirds of land plant species, including agriculturally important cereals (Smith and Read, 2008; Fitter et al., 2011). Enhancing this symbiosis has been proposed as an important tool for increasing food security and agricultural sustainability (Gosling et al., 2006; Fitter et al., 2011; Jacott et al., 2017; Thirkell et al., 2017). Whilst the host plant provides a fixed carbon (C) source for AMF, AMF transfer nutrients such as nitrogen (N) and phosphorus (P) to the plant (Hodge et al., 2001; Smith et al., 2009; Hodge and Fitter, 2010; Karasawa et al., 2012). AMF colonization affects multitrophic interactions between above- and below-ground herbivores (Yang et al., 2014) and may also enhance the uptake of silicon (Si), which can alleviate the impact of both biotic and abiotic stress (Dias et al., 2014; Garg and Bhandari, 2016; Frew et al., 2017).

The bottom-up effect of below-ground AMF on the performance of above-ground herbivores such as aphids can range from positive to negative (Gange and West, 1994; Wurst et al., 2004; Ueda et al., 2013; Simon et al., 2017; Wilkinson et al., 2019). These impacts on aphid performance likely occur because of alterations to plant defense and nutrition due to the AMF symbiosis (Wurst et al., 2004; Meir and Hunter, 2018b) and can depend on the level of AMF colonization of the host plant (Tomczak and Müller, 2017; Maurya et al., 2018; Meir and Hunter, 2018a). In turn, aphids may impose top-down effects on AMF colonization via the host plant (Babikova et al., 2014; Meir and Hunter, 2018a). Top-down and bottom-up effects can therefore modulate the outcome of each other, potentially resulting in above-below-ground multitrophic feedback loops (Meir and Hunter, 2018a). Thus, if aphids influence AMF colonization this could impact how AMF affect plant nutrient uptake and tolerance to abiotic stress in multitrophic systems. The AMF extraradical mycelium (ERM) phase is of also of key importance for interactions between plants and other rhizosphere organisms (Perotto and Bonfante, 1997; Jones et al., 2004; Hodge and Fitter, 2013) and can be directly related to AMF derived plant nutrient acquisition (Hodge et al., 2001; Barrett et al., 2011). Additionally, the ERM can be involved in plant defense, carrying signals of aphid attack to neighboring plants connected via ERM underground networks (Babikova et al., 2013). Elucidating how intra- and extraradical structures of AMF respond to top down effects is therefore important in understanding their potential for use in complex agro-ecosystems. However, current knowledge of how AMF respond to aphids sharing the same host plant is limited to the impact on AMF colonization (Babikova et al., 2014; Vannette and Hunter, 2014; Maurya et al., 2018; Meir and Hunter, 2018a).

The identity of the taxa within the AMF community colonizing the host plant can be important in determining the nutrient uptake or defense benefit gained from the symbiosis. In small, artificially selected AMF communities, AMF species identity determines the level of protection the AMF provides for the host plant against biotic stressors (Pozo et al., 2002; Sikes et al., 2009; Malik et al., 2016), and certain AMF species may deliver more or less nutrients to their host plant (Jansa et al., 2008; Leigh et al., 2009; Thirkell et al., 2016). Similarly, soil community transfer experiments suggests the AMF community structure can also be important in determining nutrient acquisition and plant growth responses (Hodge and Fitter, 2013; Williams et al., 2014; Manoharan et al., 2017; Jiang et al., 2018). Large vertebrate grazing can affect AMF communities (Ba et al., 2012; Guo et al., 2016), and insect herbivory can alter below-ground ectomycorrhizal (Gehring and Bennett, 2009), non-mycorrhizal fungi (Kostenko et al., 2012) and rhizosphere bacterial community characteristics (Kong et al., 2016), but the impact of arthropod herbivory on AMF communities is currently unknown.

The impact of herbivory on AMF structures is variable (Barto and Rillig, 2010), and phloem feeding aphids can increase or decrease the intraradical AMF colonization of their host plant (Babikova et al., 2014; Meir and Hunter, 2018a). The C limitation hypothesis proposes that above-ground removal of fixed C by herbivory will result in less C available below-ground (Wallace, 1987), although the subsequent allocation of this limited amount of C between roots and AMF contained within roots is unknown. The reduced C availability might result in changes to the AMF community because only a limited number of AMF species can be supported and thus the number and relative abundance of less competitive species might be reduced (Gange, 2007; Ba et al., 2012). Alternatively, low levels of herbivory could lead to more C allocated below-ground in an attempt for the plant to take up more nutrients for regrowth (Wamberg et al., 2003), which could increase fungal diversity (Ba et al., 2012). There are also examples of herbivory affecting the composition of ectomycorrhizal communities rather than species richness, and thus altering the beta diversity of communities, making communities more distinct (Gehring and Bennett, 2009).

Arbuscular mycorrhizal fungi communities in conventionally managed agricultural systems are often distinct from AMF communities in other settings and often have low diversity due to tillage, chemical fertilizer, pesticide and fungicide regimes (Jansa et al., 2002; Gosling et al., 2006; Wetzel et al., 2014; Hartmann et al., 2015; Manoharan et al., 2017). We selected a conventionally managed system to investigate agriculturally relevant AMF communities that are tolerant to such practices. Apart from AMF, other fungi also associate with plant roots, including other endophytic mutualists (Murphy et al., 2015; Lugtenberg et al., 2016). As some of these other fungi and their community composition can influence aphid performance (Hartley and Gange, 2009; Battaglia et al., 2013; Kos et al., 2015), the AMF community must be placed in the context of any changes in the wider fungal community.

Here, we investigate the impact of aphids on the below-ground fungal community with a focus on AMF given their key role as ecosystem engineers. Specifically, we tested the following hypotheses: (1) As aphids will depress plant growth and nutrient status, the AMF will benefit less from the association with the plant, and consequently AMF structures, both internal and external to the root, will be reduced. (2) Aphids will cause a reduction in both the alpha diversity and evenness of the soil communities, which results in a distinct soil fungal community composition (increased beta diversity).

## Materials and Methods

### Site Selection

Spring barley (*Hordeum vulgare* L., cultivar: Planet) was drilled into silty clay loam over chalk (Towthorpe, North Yorkshire, SE 91086 62387, GPS: 54.049412, -0.610267, elevation above sea level: 100 m) on March 15th, 2017. Average soil characteristics of the whole field were sampled on May 12th, 2017 and analyzed by NRM Laboratories (Berkshire, United Kingdom; Table 1). The field had been conventionally cropped with wheat and oats for the previous 5 years and was treated with conventional agrochemical inputs throughout the duration of this study (Table 1).

**Table 1:** Fieldsite soil chemical analyses and agrochemical inputs used throughout the study.

**Soil analyses (sampled May 12, 2017)**			
P (Olsen’s)		182 mg l^-1^	
K (Ammonium nitrate extracted)		274 mg l^-1^	
Mg (Ammonium nitrate extracted)		47 mg l^-1^	
pH		7.4	
Organic matter % (Loss on ignition)		6.8	
**Agrochemical inputs**		**Active ingredient concentration in concentrated product**	**Date of input**
Herbicides			
	Crystal 1.9 l^-ha^	60 g l^-1^ flufenacet, 300 g l^-1^ gl pendimethalin	March 15, 2017
	Duplosan 1.74 l^-ha^ + Harmony 0.1 l^-ha^	310 g l^-1^ Dichlorprop-P acid, 160 g l^-1^ MCPA acid, 130 g l^-1^ Mecoprop-P acid 40 g kg^-1^ metsulfuron-methyl, 400 g kg^-1^ thifensulfuron-methyl	May 25, 2017
	Gal-Gone 0.5 l^-ha^	200 g l^-1^ fluroxypy	June 03, 2017
	Axial 0.3 l^-ha^ + Agidor (Adjuvant) 0.1 l^-ha^	100 g l^-1^ pinoxaden 47% w/w methylated rapeseed oil	June 04, 2017
Fungicides			
	Siltra Xpro 0.4 l^-ha^	60 g l^-1^ bixafen, 200 g l^-1^ prothioconazole and N,N-Dimethyldecanamide	May 25, 2017
	Chlorothalonil 1.0 l^-ha^ + Siltra Xpro 0.4 l^-ha^		June 12, 2017
			
Plant Growth Regulators			
	Terpal 0.58 l^-ha^	395 g l^-1^ mepiquat chloride, 155 g l^-1^ 2-chloroethylphosphonic acid	June 03, 2017
Fertilizer			
	YARA N35 + 7SO3 231 kg^-ha^		March 16, 2017
	OMEX 0:10:15 623 kg^-ha^		March 31, 2017
	YARA N35 + 7 SO3 280 kg^-ha^		April 20, 2017

### Aphid Treatments

**FIGURE 1:**
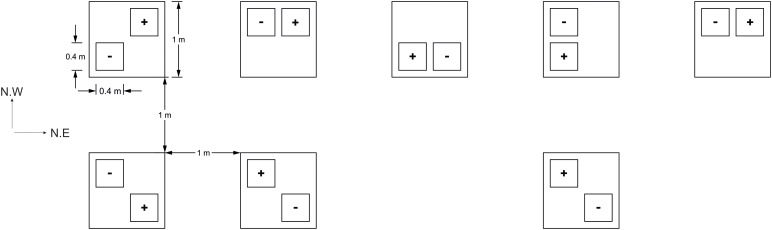
Field plot layout (3 m ×9 m). Plots of barley with (+) or without (–) aphids, *Sitobion avenae*, were arranged randomly in space within one of eight blocks (large squares) which were situated 1 m from each other. Plots were assigned a quarter of each block at random in order to reduce any positional bias within each block. The field site stood on a North West slope (NW direction of the site), and two rows were arranged perpendicular to this slope (in the North East direction; NE direction of the site).

On the April 21st, 2017, lidless and bottomless PVC boxes (40 cm × 40 cm × 25 cm) were inserted 2–3 cm below the surface of the soil around sections of developing three leaf stage seedlings, averaging 26 ± 1.7 SE plants per box. This shallow insertion, so as not to disturb plant roots, allowed an aphid impermeable seal to form between the soil and a cage structure. The interior of each box thus formed an experimental “plot.” Experimental plots were assigned to “+Aphid” or “-Aphid” treatments and arranged in a randomized block design. As the site lay on a North Western slope, plots were set out in two rows perpendicular to the slope, in a North East direction. The location of the plots in the North East and North West direction were coded as the NE and NW coordinate (respectively) of each plot within the field site. This attempted to account for any locational environmental gradients within the site, and are referred to as the NE and NW plot location hereafter (Figure 1).

**FIGURE 2:**
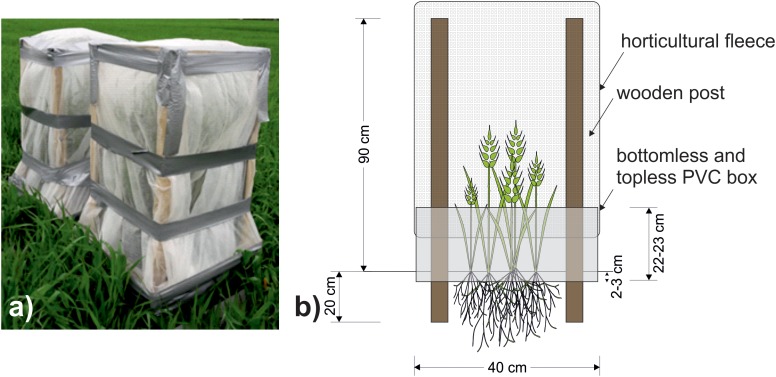
**(a)** Photograph of a pair of completed aphid cages covering plots taken on 17th May 2017. **(b)** Schematic of cage design used to prevent aphid movement between the plots. Wooden posts reaching 90 cm high were inserted 20 cm into the ground to support a cage of polypropylene horticultural fleece sealed to a 40 cm × 40 cm PVC box reaching 22–23 cm above ground.

One week later (April 28th, 2017) cages were constructed to cover all plots. The frames of the cage consisted of wooden posts inserted 20 cm into the soil and were attached to the interior of the PVC box. The cage extended 90 cm above the soil and was covered with polypropylene horticultural fleece (Figure 2), which intercepted c. 14% of the photosynthetically active radiation. English grain aphids (*Sitobion avenae*) (a single genotype, originally supplied by Koppert, Holland) were cultured on barley plants (cultivar Quench) at 20°C. From these cultures, ten 4th instar adults were taken at random and added to each +Aphid plot. All experimental plots (including -Aphid) were sealed with cages. *S. avenae* populations usually peak in the late summer months (Blackman and Eastop, 2000), however, aphids and cages were added to the crop in the current study earlier than this to stop the natural ingress of aphids into the plots. Initially, eight replicates of each treatment were set up, although one +Aphid replicate was discarded during the study due to damage to the cage caused by high wind speeds and so *N* = 8 for -Aphid treatments and *N* = 7 for +Aphid treatments. Cages remained over all treatments for the duration of the experiment, and may have reduced direct contact of some of the agrochemical inputs (see Table 1) with the plots after this date.

### Harvest

Plots were harvested on the June 13th, 2017, 46 days post-aphid addition. The number of aphids on five tillers chosen at random within each plot were recorded, and plots were dug out to 20 cm soil depth before storage at 4°C overnight. Three soil cores at each plot location were taken with a 2 cm diameter cheese corer between 20 and 30 cm soil depth. These technical replicates were processed separately to assess for the fungal hyphal length density (HLD) from each core (Hodge, 2001) but then the resulting data was pooled to create a biological replicate. After storage, the aphids were washed from any above-ground material and plant roots were separated from the soil and washed whilst still attached to the above-ground biomass. Only those roots visibly attached to a plant were stored at -20°C for DNA extraction or in 40% ethanol for the staining of fungal structures. The above-ground plant material was oven dried at 70°C for at least 96 h and the total number of plants, tillers and fertile tillers were recorded. The above-ground plant material for each plot was separated into a combined stem and leaf fraction (henceforth referred to as stem material) as well as a separate grain fraction. It should be noted that due to the size of the stem and leaf material, the stem and leaf fraction was weighed to the nearest gram. These fractions were homogenized in a kitchen blender (Igenix ig 8330, Ipswich, United Kingdom) before ball milling to a fine powder (Retsch MM400, Retsch GmbH, Haan, Germany). The resulting material was then analyzed for C and N ratios via a Elementar Vario El Cube (Elementar UK, Ltd., Stockport, United Kingdom) and pelleted for X-ray fluorescence (XRF) analysis (Thermo Fisher Scientific^TM^ portable X-ray fluorescence analyzer) to determine P and Si concentrations as described by Reidinger et al. (2012).

The roots stored in 40% ethanol were stained for fungal structures via the acetic acid–ink staining method (Vierheilig et al., 1998), modified as in Wilkinson et al. (2019). Structures were assessed under a Nikon 50i eclipse microscope (Nikon UK Ltd., Surrey, United Kingdom) under 200 X magnification. As fungi other than AMF colonize plant roots in natural systems, a method that calculates both the most conservative estimation of AMF root length colonization (RLC) (RLC min) and least conservative (RLC max) was employed (Brundrett et al., 1994).

### DNA Extraction, PCRs, and Sequencing

Frozen root material was lyophilized for 36 h and ball milled to a fine powder. The DNA was extracted from this material using a DNeasy PowerPlant Pro Kit (QIAGEN N.V, Venlo, Netherlands) according to the manufacturer’s instructions with the exception that, in order to increase the DNA yield, the DNA solution was eluted twice through the membrane in the final step. DNA concentrations were assessed (NanoDrop^TM^ 8000 Spectrophotometer Thermo Fisher Scientific) and diluted to 20 ng μl^-1^ before the PCR analysis.

Two regions (amplicons) of fungal ribosomal DNA were amplified via PCR (Table 2); an amplicon that captures the diversity of the entire fungal community (total fungi), and an amplicon that captures AMF specific diversity at a higher resolution and species coverage (AMF specific). After initial amplification via primary PCRs, secondary PCRs attached illumina sequencing barcodes. For all PCRs the reaction consisted of 0.5 μl DNA, 0.1 μl of forward and reverse primers (20 mM) and 12.5 μl BioMix Red (Bioline, London, United Kingdom) made up to 25 μl reaction volume with molecular grade dH_2_O. PCRs were carried out using a T100^TM^ Thermal Cycler (BioRAD, Hercules, CA, United States). The PCR products of the secondary PCRs were purified using a QIAquick PCR Purification Kit (Qiagen) and the purified concentrations were measured using a Qubit^®^3.0 fluorometer (Thermo Fisher Scientific^TM^, Waltham, MA, United States). To mix the products of the two amplicons at equimolar concentrations to reduce sequencing depth bias during simultaneous DNA sequencing, these products were lyophilized overnight and re-suspended in molecular grade H_2_O to achieve desired concentrations. Quality control and library preparation was carried out by the University of York Bioscience Technology Facility, and the resulting samples were sequenced using an illumina MiSeq system (illumina, San Diego, CA, United States) at 2 ∼ 300 bp: Briefly, unique barcode sequences (Nextera XT index primers, illumina) were added onto amplicons tagged with illumina adapter sequences via PCR. Amplicons were then purified and pooled at equimolar ratios and then diluted and denatured. Samples were spiked with PhiX library spikes (illumina) for added sequence variety to enhance the distinguishing of fluorescent signals of clusters during sequencing. Samples were run using the MiSeq 600 cycle kit (illumina).

**Table 2:** Primer sets and PCR conditions used in nested PCRs for “total fungi” and “AMF specific” amplicon sequencing.

Amplicon		Primer pairs	Cycling conditions	DNA used in reaction
**Total fungi**				
	Primary PCR	ITS1F (Gardes and Bruns, 1993) to ITS4 (White et al., 1990)	5 min @ 95°C; 35 cycles (30 s @ 94°C, 45 s @ 55°C, 90 s @ 72°C); 10 min @ 72°C.	10 ng extracted DNA
	Secondary PCR (illumina tagged primers)	GITS7 (Ihrmark et al., 2012) to ITS4 (White et al., 1990)	5 min @ 95°C; 30 cycles (30 s @ 94°C, 45 s @ 55°C, 90 s @ 72°C); 5 min @ 72°C.	Total fungi primary PCR product (diluted 1:1000)
**AMF specific**				
	Primary PCR	AML1 to AML2 (Lee et al., 2008)	2 min @ 95°C; 30 cycles (30 s @ 94°C, 30 s @ 59°C, 90 s @ 72°C), 10 min @ 72°C.	10 ng extracted DNA
	Secondary PCR (illumina tagged primers)	WANDA (Dumbrell et al., 2011) to AML2 (Lee et al., 2008)	5 min @ 95°C; 30 cycles (30 s @94°C, 40 s @ 59°C, 90 s @ 72°C), 10 min @ 72°C.	AMF specific primary PCR product (undiluted)

### Bioinformatic Analysis

The raw forward and reverse reads were merged together resulting in a total of *c.* 1.5 million reads which were processed using QIIME2^1^ (Caporaso et al., 2010). Reads were stripped of their primer and barcoding sequences and untrimmed reads were discarded (0.9%). Read quality of the merged reads were estimated using eestats2 and reads from AMF specific amplicons were truncated to 270 bp from the front end due to estimated sequence quality drop off at this point, whilst total fungal amplicons were not truncated. Reads were then dereplicated and clustered into operational taxonomic units (OTUs) using Usearch10 (Edgar, 2010) with 97% similarity. The resulting 517 total fungi amplicon OTUs were BLASTed against the UNITE ITS (Kõljalg et al., 2013) database and eight non-fungal OTUs were removed, resulting in 509 total fungi OTUs, with the total read number per sample ranging from 53,333 to 129,338. Samples were normalized to 70,000 reads per sample using Usearch10’s “norm” function. Identical virtual taxon accessions according to the UNITE database were merged together yielding the 155 total fungi amplicon OTUs used in subsequent analysis.

A total of 27 OTUs were identified for the AMF specific amplicon. These were BLASTed against the maarjAM database (Öpik et al., 2010) accessed on 13th June 2018 and five OTUs with less than 96% coverage or similarity to taxa in the database were discarded. This resulted in a total read number from 12,035 to 21,706 per sample. Reads per sample were normalized to 16,500. AMF virtual taxonomic (VT) identities were assigned to the OTUs according to the greatest BLAST coverage and similarity; where OTUs could not be assigned to a single VT, VTs were labeled as unassigned. A phylogenetic tree of the sequences was built to identify identical VT accessions. Identical VTs were merged together resulting in 15 OTUs used in subsequent analysis.

### Statistical Analysis

Alpha diversity metrics [OTU/VT richness, Shannon’s Index (e), Peilou’s Evenness and Simpson’s Diversity] were calculated using Usearch10’s (Edgar, 2010) alpha diversity command and all subsequent analyses were carried out in R version 3.3.2 (October 31, 2016) (R Core Team, 2016). To test the effect of the aphid treatment on AMF and total fungi alpha diversity metrics, AMF family relative abundance, and plant and AMF structures, aphid presence was used as an explanatory variable in a linear model, using the R packages “lme4,” “lmerTest,” “lsmeans,” and “car.” Plot location, measured as the NE and NW distance of each plot from the NW corner of the field site, was used as a covariate (see Table 3). To identify fungal OTUs/VTs whose presence predicts aphid treatment we employed indicator species analysis using the “indicspecies” package. The identification and estimation of abundances of AMF is more accurate using the AMF specific amplicon (Berruti et al., 2017), thus total fungi OTUs corresponding to AMF taxa were excluded from the indicator species analysis of the total fungi amplicon. The effect of aphid presence on community composition between samples as measured via Bray-Curtis dissimilarity (Beta diversity) was analyzed using a PERMANOVA via the “Adonis” function in the “Vegan” R package. This was visualized via non-metric multidimensional scaling (NMDS). Relationships between community composition and plant biomass and nutrition, and AMF structures as well as plot location were tested by applying the “envfit” function to the NMDS.

**Table 3:** Mean (±SE) above-ground plant biomass and nutrient concentrations, and AMF structures of experimental barley plots treated with or without aphids, using the location of the plot in the NE and NW direction of the site as a model covariate.

					Plot location covariate			
			Aphid presence		NE		NW	
	-Aphid	+Aphid	*F*_1,11_	*P*	*F*_1,11_	*P*	*F*_1,11_	*P*
Plant No m^-2 1.^	172.5 ± 20	156.9 ± 11.3	0.439	0.521	0.085	0.777	0.764	0.401
Fertile tiller No m^-2^	301.9 ± 29.4	319.4 ± 36.3	0.164	0.693	0.353	0.565	0.647	0.438
Total tiller No m^-2^	446.3 ± 28.1	418.8 ± 31.3	0.440	0.521	1.99	0.186	0.460	0.512
Stem DW m^-2 2.^	356 ± 29.4	356 ± 21.9	0.002	0.963	0.097	0.761	1.731	0.215
Plot grain DW m^-2^	68.1 ± 8.6	67.9 ± 2.9	<0.001	0.982	0.244	0.631	0.024	0.880
Mean tiller DW	0.95 ± 0.06	1.02 ± 0.05	0.985	0.342	1.706	0.218	0.767	0.399
Mean grain DW	0.22 ± 0.01	0.23 ± 0.03	0.007	0.934	0.182	0.678	0.967	0.347
Stem [P]^3.^	2.46 ± 0.10	2.46 ± 0.14	<0.001	0.996	0.030	0.865	0.040	0.845
Stem [N]	18.16 ± 0.71	19.08 ± 0.89	0.664	0.432	0.139	0.716	0.035	0.855
Stem [Si]	9.69 ± 0.70	8.57 ± 0.48	1.599	0.232	0.005	0.943	0.077	0.786
Stem [C]	420.9 ± 2.13	420.1 ± 2.16	0.055	0.819	0.370	0.556	<0.001	0.979
Stem C:N	23.38 ± 1.06	22.28 ± 0.97	0.574	0.465	0.343	0.570	0.009	0.926
Stem N:P	7.40 ± 0.11	7.80 ± 0.18	5.143	**0.045**	2.806	0.122	1.200	0.297
Grain [P]	3.63 ± 0.14	3.49 ± 0.10	0.899	0.363	5.122	**0.045**	0.059	0.813
Grain [N]	17.50 ± 0.52	18.38 ± 0.43	1.734	0.215	0.719	0.415	0.154	0.702
Grain [Si]	10.30 ± 0.55	9.43 ± 0.58	1.239	0.289	0.443	0.519	0.031	0.864
Grain [C]	418.7 ± 1.89	421.7 ± 1.74	1.324	0.274	0.194	0.668	0.025	0.876
Grain C:N	24.05 ± 0.72	23.00 ± 0.62	1.194	0.298	0.442	0.520	0.102	0.756
Grain N:P	4.85 ± 0.21	5.28 ± 0.16	4.736	0.052**⋅**	10.591	**0.008**	0.376	0.552
RLC Min^4.^	32.67 ± 3.14	33.48 ± 3.06	0.034	0.857	0.021	0.888	0.409	0.536
RLC Max^4.^	47.45 ± 3.71	49.91 ± 3.91	0.284	0.605	0.149	0.707	0.715	0.416
HLD^5.^	0.32 ± 0.03	0.34 ± 0.04	0.315	0.586	0.026	0.875	0.001	0.973
Arbuscule^4.^	29.20 ± 2.57	29.77 ± 3.56	0.019	0.893	0.139	0.716	1.078	0.322
Vesicle^4.^	2.75 ± 1.03	5.29 ± 1.78	3.296	0.097**⋅**	0.021	0.888	11.606	**0.006**

*P-values highlighted in bold are significant at a value of <0.05, P-values highlighted with **⋅** are >0.05 and <0.1. ^1^No., Number; ^2^DW, dry weight (g); ^3^[], concentration (mg g^-1^); ^4^RLC, AMF root length colonization where Min, most conservative estimate; Max, least conservative estimate; RLC, arbuscule and vesicle values are % of root length; ^5^HLD, hyphal length density (m hyphae g^-1^ soil).*

## Results

### Effect of Aphid Presence on Plant Nutrition and Biomass, and AMF Structures

The mean number of aphids *per tiller* ± SE in “+Aphid” plots was 24.9 ± 8.2, and ranged from between 1.8 and 75, whilst no aphids were present on any tillers investigated for “-Aphid” plots. Whilst the majority of plant biomass and nutrition, and AMF physiological traits were not influenced by aphid treatment (Table 3), aphid treatment significantly increased the stem N:P ratio by 5.4% (Table 3), and there was a near significant increase of 8.9% (*P* = 0.052) of the grain N:P ratio. Grain P concentration and N:P ratio was associated with the position of the plot in the NE direction of the site (Table 3). While aphid treatment did not significantly impact any AMF structures, there was a trend that the vesicle frequency almost doubled in the roots of plants hosting aphids (*P* = 0.097). The frequency of vesicles was associated with the position of the plot in the NW direction (Table 3).

### Effect of Aphid Presence on Total Fungal and AMF Communities in the Root

Within the entire fungal community in plant roots across both aphid treatments, 153 “total fungi” OTUs were identified from nine fungal phyla and 2 OTUs that could not be assigned at the phylum level. The highest abundance of sequences were assigned to Ascomycota (90.2%), followed by Basidiomycota (5.3%), unclassified fungi (3.5%), Glomeromycota (0.83%), and Chytridiomycota (0.08%). In contrast, sequences from Rozellomycota, Mortierellomycota, Entomophthoromycota, Mucoromycota, and Zoopagomycota contributed less than a combined 0.1% of sequence abundance. Within the AMF specific amplicon, 12 OTUs were assigned to VTs whilst three OTUs could not be assigned to a singular VT (see Supplementary Table S1). These VTs belonged to seven AMF families: Glomeraceae (6), Paraglomeraceae (1), Diversisporaceae (2), Ambisporaceae (1), Gigasporaceae (1), Archaeosporaceae (3), and Acaulosporaceae (1).

Aphid presence did not affect the species richness within the entire fungal community, but did increase its evenness (Table 4). Aphid presence also did not affect any AMF specific alpha diversity metrics. The Simpson’s diversity of the AMF specific community was linked to plot location in the NE direction of the field site (Table 4).

**Table 4:** Mean (±SE) Alpha diversity metrics for “total fungi” amplicon OTUs, and “AMF specific” amplicon VTs from barley plots without or without the presence of aphids, using the location of the plot in the NE and NW direction of the site as a model covariate.

						Plot location covariate			
				Aphid presence		NE		NW	
		-Aphid	+Aphid	*F*_1,11_	*P*	*F*_1,11_	*P*	*F*_1,11_	*P*
**Total fungi**									
OTU richness		92.6 ± 2.5	89.4 ± 2.3	0.889	0.366	0.281	0.607	0.087	0.774
Shannon’s index		2.54 ± 0.09	2.75 ± 0.06	3.725	0.080**⋅**	0.008	0.932	0.170	0.688
Peilou’s evenness		0.56 ± 0.02	0.61 ± 0.01	5.090	**0.045**	<0.001	0.977	0.260	0.620
Simpson’s diversity		0.15 ± 0.02	0.11 ± 0.01	3.059	0.108	0.527	0.483	0.377	0.551
**AMF**									
VT richness		11.3 ± 0.3	11.1 ± 0.5	0.033	0.859	0.004	0.953	0.105	0.752
Shannon’s index		1.17 ± 0.11	1.20 ± 0.09	0.047	0.833	2.646	0.132	0.264	0.618
Peilou’s evenness		0.49 ± 0.04	0.51 ± 0.04	0.075	0.790	3.018	0.110	0.429	0.526
Simpson’s diversity		0.47 ± 0.05	0.44 ± 0.05	0.226	0.644	6.207	**0.030**	0.176	0.683

*Richness: number of OTUs/VTs. P-values highlighted in bold are significant at a value of <0.05, P-values highlighted with **⋅** are >0.05 and <0.1.*

Aphid presence had no effect on the relative abundance of AMF reads within the entire fungal community. Within the AMF specific amplicon the relative abundance of the Gigasporaceae family tended to increase when aphids were present (Table 5). The relative abundance of AMF reads within the entire fungal community, and the relative abundance of the Gigasporaceae and Ambisporaceae families present in the AMF specific community were associated with the location of the plot in the NE direction of the site (Table 6).

**Table 5:** Mean (±SE) relative abundances of the AMF sequences within the “total fungi” amplicon, and of AMF family sequences from the “AMF specific amplicon,” from experimental barley plots infested with or without aphids, using the location of the plot in the NE and NW direction of the site as a model covariate.

					Plot location covariate			
			Aphid presence		NE		NW	
Relative abundance	-Aphid	+Aphid	*F*_1,11_	*P*	*F*_1,11_	*P*	*F*_1,11_	*P*
AMF	0.53 ± 0.29	1.18 ± 0.63	1.450	0.254	5.447	**0.040**	0.145	0.710
Acaulosporaceae	0.02 ± 0.02	0	–	–	–	–	–	–
Ambisporaceae	3.21 ± 0.79	2.92 ± 0.71	0.123	0.732	8.111	**0.016**	0.036	0.854
Archaeosporaceae	1.23 ± 0.41	0.63 ± 0.19	1.828	0.204	0.056	0.817	1.848	0.201
Diversisporaceae	3.72 ± 0.68	5.68 ± 2.52	0.767	0.400	2.241	0.163	0.198	0.665
Gigasporaceae	0.77 ± 0.20	1.48 ± 0.52	4.106	0.067**⋅**	13.560	**0.004**	0.553	0.473
Glomeraceae	85.44 ± 2.18	83.43 ± 2.30	0.420	0.530	0.778	0.397	<0.001	0.990
Paraglomeraceae	5.62 ± 1.32	5.86 ± 1.54	0.016	0.900	0.658	0.434	1.119	0.313

*P-values highlighted in bold are significant at a value of <0.05, P-values highlighted with **⋅** are >0.05 and <0.1.*

**Table 6:** Indicator species analysis of AMF specific amplicon VTs and total fungi amplicon OTUs as indicators of untreated or aphid treated barley plots.

	OTU/VT	VT/OTU present within treatment		IndVal	*P*
		-Aphid	+Aphid		
**AMF specific**					
Acaulospora VTX00030	✓	×	0.35	1.000
Glomus VTX00199	×	✓	0.49	0.349
**Total fungi**					
Ascomycota; Pseudeurotiaceae	✓	×	0.92	**0.006**
Ascomycota; Helotiales	✓	×	0.88	**0.046**
Ascomycota; Halosphaeriaceae	✓	×	0.87	**0.032**
Basidiomycota; Cystobasidiaceae	×	✓	0.81	**0.029**

*Only total fungi amplicon OTUs which significantly indicate (bold) the aphid treatments are shown. ✓ indicates the presence of the taxon, while × indicates its absence.*

All but two AMF specific amplicon VTs were found in both aphid and no aphid treatments, however, the exceptions were not strong indicators of aphid presence or absence (Table 6). Several Ascomycota taxa were strong predictors of the absence of aphids for the total fungi amplicon, whilst a member of the family Cystobasidiaceae indicated aphid presence.

**FIGURE 3:**
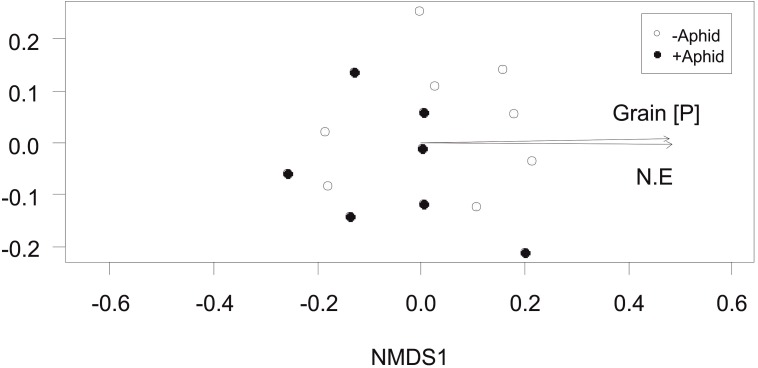
Total fungi community composition: Non-Metric Multidimensional Scaling (NMDS) of total fungi community distribution based on Bray-Curtis dissimilarity (stress = 0.158) obtained from the abundance of “total fungi amplicon” OTUs under “+Aphid,” or “–Aphid” treatments. Total fungi community composition was significantly associated with the grain P concentration (mg g^-1^) in the plots (Grain [P]) and the location of the plot in the NE direction of the site (NE).

The total fungi amplicon community composition between plots, measured as Bray-Curtis dissimilarity (beta diversity) was not significantly affected by aphid presence (PERMANOVA: *F*_1,13_ = 1.75, *P* = 0.131). However, community composition correlated with plot location in the NE direction of the site (*R*^2^ = 0.46, *P* = 0.031) and grain P concentration (*R*^2^ = 0.46, *P* = 0.021; Figure 3).

**FIGURE 4:**
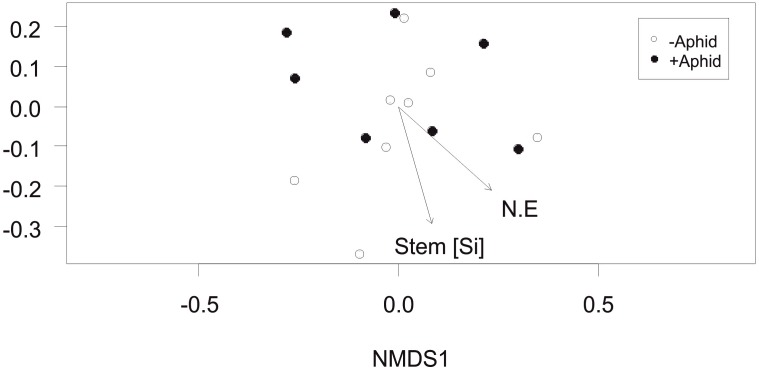
Arbuscular mycorrhizal fungi specific community composition: Non-Metric Multidimensional Scaling (NMDS) of the AMF community distribution based on Bray-Curtis dissimilarity (stress = 0.154) obtained from the abundance of “AMF specific amplicon” VTs under “+Aphid” or “–Aphid” treatments. AMF specific community composition was significantly associated with the barley stem silicon Si (mg g^-1^) concentration in the plots (Stem [Si]), and the location of the plot in the NE direction of the site (NE coordinate).

The community composition of AMF specific VTs in the roots of barley plants between plots was not affected by the presence of aphids (*F*_1,13_ = 0.46, *P* = 0.604). However, the environmental factors of stem Si concentration and location of the plots in the NE direction of the field site were significantly correlated with the community composition (*R*^2^ = 0.39, *P* = 0.049 and *R*^2^ = 0.40, *P* = 0.049, respectively; Figure 4).

## Discussion

This study aimed to investigate the impact of aphids on soil fungi in a conventionally managed agricultural system. It was hypothesized that by supressing plant nutrition and growth, aphid feeding would lead to negative impacts on AMF structures and species richness, as well as the evenness of the AMF and other fungi communities, measured here through analysis of an AMF specific amplicon and another less precise, but wider encompassing amplicon (total fungi). It was also proposed that the effects of aphids would impact the compositions of these communities (Gehring and Bennett, 2009), and the relative abundance of AMF taxa within them. In fact, aphid presence had less of an impact on AMF community structure than the location of the host plant in the field, although there was a trend of the abundance of AMF vesicles, and the abundance of the Gigasporaceae family to increase when aphids fed on the host plant. Within the total fungi community, the relative abundance of AMF was also affected by location rather than aphid presence. However, aphid presence increased evenness across the total fungi community.

### Effects of Aphids on the Plant Biomass and Nutrition, and AMF Structures

Contrary to our hypothesis, *S. avenae* had little effect on the above-ground nutrition of barley in the field, although aphid presence tended to increase the above-ground plant N:P ratio (Table 3), possibly due to nutrient re-allocation caused by aphid feeding (Sandstrom et al., 2000; Thompson and Goggin, 2006; Nowak and Komor, 2010), or differences between the requirement for N and P by aphids (Tao and Hunter, 2012). Moreover, aphid presence did not reduce above-ground biomass in the present study. Since the aphids used in this experiment were cultured under controlled conditions it is likely that as the aphids did not vector any plant viruses that are a major contributor to aphid related yield loss in cereals (McKirdy et al., 2002). While it is possible that the agro-chemical inputs of fungicides, herbicides and plant growth regulators in this conventionally managed system may have influenced aphid development, the total number of aphids per tiller, and thus, estimated in each “+ Aphid” plot, remained high. However, the aphid population in the current experiment was launched at an earlier date than in non-controlled systems (Blackman and Eastop, 2000) which may have also influenced the results. AMF colonization of barley roots was high compared to that measured in field studies previously (Boyetchko and Tewari, 1995), and in certain glasshouse studies (Grace et al., 2009). Thus, while we originally hypothesized that aphids would have a negative impact on above-ground plant biomass and nutrition which in turn, would reduce both the internal and external phases of the AMF, no negative impact occurred. This may explain why both AMF RLC and hyphal length density were not affected by the presence of aphids in this study. However, there was a trend for aphids to increase the proportion of vesicles in plant roots. Mechanical defoliation has also been shown to influence the proportion of vesicles in plant roots when grown with a native AMF soil community (Garcia and Mendoza, 2012). As vesicles are lipid storage organs in AMF, and AMF derive lipids from the host plant (Keymer and Gutjahr, 2018), this might suggest that more fixed C is available to the AMF via the plant under aphid herbivory. Alternatively, AMF vesicle numbers may have increased in a response to less C flow from the plant in order to aid survival under stressed conditions, similar to the AMF response observed in cool conditions (Hawkes et al., 2008), It is also possible that the frequency of AMF with a propensity to form more vesicles occurred *within* the Glomeracea community. However, the tendency for aphid presence to increase the relative abundance of the AMF family Gigasporaceae, which do not develop vesicles (Smith and Read, 2008), suggests that this explanation is less likely.

### Effects of Aphids on AMF and Total Fungal Communities

The second hypothesis proposed that the effects of aphid feeding on the host plant would reduce AMF and total fungal species richness and evenness. The low richness of AMF species identified in the current study is similar to that documented in other conventionally managed barley monoculture systems (Manoharan et al., 2017), and as the read depth achieved for the AMF specific amplicon is sufficient to capture AMF diversity (Vasar et al., 2017), it can be assumed that this is an accurate representation. The number of species of AMF were not impacted by aphid presence perhaps as there was no effect of aphid presence on above-ground plant biomass; however, this is similar to the lack of an effect of arthropod feeding on pinyon pine associated ectomycorrhizal communities (Gehring and Bennett, 2009). This was also reflected in the number of species in the entire fungal community in the current study. Moreover, and counter to expectations, aphid presence increased the evenness of the entire fungal community in the present study. Nutrient flows below-ground were not measured in the current study, but aphids can affect below-ground respiration depending on plant growth stage, potentially due to alterations in C availability to soil microbes (Vestergård et al., 2004). Aphids can also alter the profiles of compounds, released from plant roots (Hoysted et al., 2018) and can also change the profiles of sugars found in AMF hyphae sharing the same host plant (Cabral et al., 2018). Moreover, aphids excrete honeydew as a result of their C rich diet of phloem sap which can be utilized as a C source by soil microbiota, thus shaping community structure and biomass (Katayama et al., 2014; Milcu et al., 2015). As more C sources become available in the root, it is possible that niches may enlarge allowing less competitive fungi to compete, reducing the dominance of abundant taxa. However, it should be noted that aphid induced alterations to soil organisms can occur independently of honeydew C inputs (Sinka et al., 2009), and that soil microbes can be influenced by aphid induced changes to plant root exudates in systems where honeydew does not reach the microbe (Kim et al., 2016). Above-ground herbivory generally stimulates the cycling of nutrients by decomposers in the soil (A’Bear et al., 2014), which could partly explain increases in the N:P ratios of plant tissues infested by aphids in the current study.

It was hypothesized that the abundance of AMF taxa within the AMF community would be impacted by aphid presence, and there was a marginal increase in the abundance of Gigasporaceae under aphid infestation of the host plant. A recent meta-analysis revealed that members of this family are tolerant to fertilizer input disturbances, suggesting a role aside from nutrient acquisition, perhaps in plant defense (van der Heyde et al., 2017b). Species indicator analysis may also identify taxa affected by treatments, however, as low abundance taxa typically score as poor indicators, the results of this method may differ from those investigating relative abundances (Longa et al., 2017). None of the AMF VTs were indicators of either treatment, but several total fungi amplicon OTUs were significant indicators of aphid presence or absence. Currently, it is unclear whether these fungi are responding to changes in nutrient availability, whether the plant recruits them in response to aphid feeding to aid with defense (Kong et al., 2016; Pineda et al., 2017), or whether the recruitment of specific soil microbes ultimately benefits the aphid (Kim et al., 2016).

No clear effects of aphid presence were found on community composition, perhaps as a longer period of top down pressure is required to impact this metric. For example, the effects of grazing by large vertebrates on AMF community structure are strongly linked to the length of the grazing (van der Heyde et al., 2017a). However, as plant communities are removed regularly in cereal systems, and aphid feeding is seasonal (Blackman and Eastop, 2000), only a relatively short window is available for these interactions to occur. Plot location, grain P concentration and AMF abundance as well as community composition were tightly linked in the current study. AMF communities may be associated with environmental and nutritional gradients in soil systems (Bainard et al., 2014; Horn et al., 2014; Guo et al., 2016; Yao et al., 2018) which also likely reflects the spatial differences measured in above-ground plant nutrition and AMF physiology here. Although care was taken to reduce the spread of plots across the slope of the site, these spatial differences highlight the importance of environmental heterogeneity within relatively small distances (less than 10 m) in field sites, and this could have contributed to masking top down effects of aphids on community composition in the current study. Whether the associations between AMF community composition and plant Si are due to AMF uptake of Si (Garg and Bhandari, 2016; Frew et al., 2017), or an artifact of AMF responses to soil pH gradients or water availability (Bainard et al., 2014) requires further study.

## Conclusion

Aphids increased the evenness of the entire fungal community within plant roots, and also tended to increase the level of vesicles and abundance of the AMF family Gigasporaceae. Whether these increases are due to increased C allocation below-ground by plants attempting to increase nutrient uptake, or the active selection of fungal taxa in response to herbivory requires elucidation. Whether these changes in the below-ground soil community feed back into altered aphid performance is currently unclear, but the response of agriculturally relevant fungal communities to top-down effects of herbivory suggests that above-below-ground community feedback could occur in agricultural systems.

## Data Availability

The raw sequence datasets for this study are available in the European Nucleotide Archive accession number PRJEB31469. The datasets for this study are available in the Dryad digital depository (doi: 10.5061/dryad.864pt1f/1).

## Author Contributions

TW, AH, SH, and JF contributed to the experimental design of the study. TW conducted the experiments and analyzed the data with consultation of the bioinformatics analysis from J-PM. All authors contributed to the writing of the manuscript.

## Conflict of Interest Statement

The authors declare that the research was conducted in the absence of any commercial or financial relationships that could be construed as a potential conflict of interest.

## Footnotes

1 https://qiime2.org
