# Supplementary material for: Aphids Influence Soil Fungal Communities in Conventional Agricultural Systems
Source: Front Plant Sci. 2019 Jul 12;10:895. doi: 10.3389/fpls.2019.00895 (PMC6640087; doi:10.3389/fpls.2019.00895)
Supplement: Supplementary file 1 [file Table_1.docx]

Supplementary Material for

Aphids influence soil fungal communities in conventional agricultural systems

Thomas D.J. Wilkinson^1,3^, Jean-Pascal Miranda^1^, Julia Ferrari^1*^, Sue E. Hartley^1,2^ and Angela Hodge^1^

^1^Department of Biology, University of York, Wentworth Way, York, YO10 5DD, U.K.

^2^York Environmental Sustainability Institute, University of York, YO10 5DD, U.K.

**Current address: ^3^ADAS Gleadthorpe, Meden Vale, Mansfield NG20 9PD, U.K.**

Table S1. AMF VT (Virtual taxa) identified via AMF specific amplicon sequencing. Unassigned = could not be assigned to a singular VT.

| Family | Species | VT |
| --- | --- | --- |
| Acaulosporaceae | Acaulospora sp. | VTX00030 |
| Ambisporaceae | Ambispora sp. | VTX00283 |
| Archaeosporaceae | Archaeospora sp. | unassigned |
| Archaeosporaceae | Archaeospora sp. | VTX00245 |
| Archaeosporaceae | Archaeospora sp. | VTX00338 |
| Diversisporaceae | Diversispora sp. | unassigned |
| Diversisporaceae | Diversispora sp. | VTX00354 |
| Gigasporaceae | Scutellospora sp. | VTX00052 |
| Glomeraceae | Glomus sp | unassigned |
| Glomeraceae | Glomus sp. | VTX00064 |
| Glomeraceae | Glomus sp. | VTX00065 |
| Glomeraceae | Glomus sp. | VTX00105 |
| Glomeraceae | Glomus sp. | VTX00143 |
| Glomeraceae | Glomus sp. | VTX00199 |
| Paraglomeraceae | Paraglomus sp. | VTX00281 |
